# Perceived Comparative Harm of Cigarettes and Electronic Nicotine Delivery Systems

**DOI:** 10.1001/jamanetworkopen.2019.15680

**Published:** 2019-11-20

**Authors:** Amy L. Nyman, Jidong Huang, Scott R. Weaver, Michael P. Eriksen

**Affiliations:** 1 Georgia State University School of Public Health, Atlanta; 2 Department of Health Policy and Behavioral Sciences, Georgia State University School of Public Health, Atlanta; 3 Department of Population Health Sciences, Georgia State University School of Public Health, Atlanta

## Abstract

This survey study examines the changing attitudes regarding the perceived harm of electronic nicotine delivery systems (ENDS) among smokers, nonsmokers, and ENDS users from 2017 to 2018.

## Introduction

Scientific evidence on the health risks associated with using electronic nicotine delivery systems (ENDS) is rapidly evolving. Perceived harm of ENDS is also changing rapidly. The percentage of US adults, including current smokers, who perceive using ENDS as equally or more harmful than smoking cigarettes increased between 2012 and 2015, with rates stable through 2017.^1^ Comparative harm perceptions are important factors associated with smokers’ decisions to use ENDS^2,3^ and have implications for population health. This study uses data from late 2018 to assess changes in US adults’ comparative harm perceptions of ENDS and cigarettes after a year characterized by intense public debate and regulatory efforts to restrict youth access to ENDS.

## Methods

This survey study was approved by the institutional review board at Georgia State University with a waiver of informed consent because the study posed minimal risk to participants, data were deidentified, and the KnowledgePanel^4^ invitation to participate in the study contained some elements of informed consent. We followed the American Association for Public Opinion Research (AAPOR) reporting guideline.

Data were obtained from the Tobacco Products and Risk Perceptions Surveys conducted in August through September 2017 and November through December 2018 by the Georgia State University Tobacco Center of Regulatory Science. The Tobacco Products and Risk Perceptions Survey is an annual cross-sectional survey of a probability sample and representative oversample of preidentified cigarette smokers drawn from KnowledgePanel,^4^ a web panel designed to be representative of noninstitutionalized US adults 18 years and older. In total, 5992 US adults completed the 2017 survey (cumulative response rate, 5.8%) and 5989 US adults completed the 2018 survey (cumulative response rate, 6.0%). Study-specific poststratification weights, based on demographic and geographic distributions from the Current Population Surveys as benchmarks for adjustment, were computed using an iterative proportional fitting procedure adjusting for survey nonresponse. Comparative perceived harm of ENDS vs cigarettes was asked of those aware of ENDS and was measured as follows: “Is using electronic vapor products less harmful, about the same, or more harmful than smoking regular cigarettes?” Response options were much less harmful, less harmful, about the same level of harm, more harmful, much more harmful, and I don’t know. Weighted point estimates and 95% CIs were obtained, and differences between 2017 and 2018 data were assessed by χ^2^ tests of association. Data analyses were conducted using SPSS statistical software Complex Samples module version 25 (IBM). *P *values were 2-sided, and statistical significance was set at less than .05. Analyses were conducted in May and June 2019.

## Results

There were 5357 participants (mean [SD] age, 47.5 [17.1] years; 2660 [49.7%] women) in our 2017 sample and 5445 participants (mean [SD] age, 48.0 [17.2] years; 2849 [52.3%] women) in our 2018 sample. After study-specific poststratification weights were included, the 2017 sample included 51.0% women and the 2018 sample included 50.9% women. Between 2017 and 2018, the percentage of US adults who perceived ENDS to be less harmful than cigarettes decreased (29.3% [95% CI, 27.8%-30.7%] vs 25.8% [95% CI, 24.4%-27.2%]), while there was an increase in the proportion of US adults who perceived ENDS to be more harmful (2.4% [95% CI, 2.0%-2.9%] vs 4.4% [95% CI, 3.9%-5.1%]) or much more harmful (1.9% [95% CI, 1.5%-2.5%] vs 3.7% [95% CI, 3.1%-4.4%]) (Table). Between 2017 and 2018, ENDS were increasingly perceived to be much more harmful among current smokers (1.8% [95% CI, 1.1%-2.9%] vs 4.4% [95% CI, 3.1%-6.4%]) and former smokers (1.0% [95% CI, 0.5%-1.9%] vs 3.5% [95% CI, 2.5%-4.8%]). The percentage of adults perceiving ENDS to be equally as harmful as cigarettes also increased from 36.4% (95% CI, 34.9%-38.0%) to 43.0% (95% CI, 41.5%-44.6%), while the percentage of adults uncertain of the comparative harm decreased from 25.3% (95% CI, 24.0%-26.7%) to 19.3% (95% CI, 18.0%-20.5%).

**Table.** Perceived Harm of ENDS Relative to Combustible Cigarettes Among US Adults in 2017 and 2018

Smoking Status	Respondents, % (95% CI)^a^		*P* Value^b^
	2017	2018	
Overall respondents, No.^c^	5357	5445	
Much less harmful	4.7 (4.0-5.4)	3.8 (3.2-4.5)	<.001
Less harmful	29.3 (27.8-30.7)	25.8 (24.4-27.2)	
About the same level	36.4 (34.9-38.0)	43.0 (41.5-44.6)	
More harmful	2.4 (2.0-2.9)	4.4 (3.9-5.1)	
Much more harmful	1.9 (1.5-2.5)	3.7 (3.1-4.4)	
Do not know	25.3 (24.0-26.7)	19.3 (18.0-20.5)	
Never smokers, No.	2653	2801	
Much less harmful	3.6 (2.8-4.6)	2.3 (1.7-3.0)	<.001
Less harmful	31.3 (29.3-33.4)	27.7 (25.9-29.7)	
About the same level	37.4 (35.3-39.6)	44.7 (42.6-46.8)	
More harmful	1.9 (1.3-2.6)	4.8 (4.0-5.8)	
Much more harmful	2.5 (1.9-3.3)	3.7 (2.9-4.6)	
Do not know	23.4 (21.6-25.3)	16.8 (15.3-18.4)	
Former smokers, No.	1533	1505	
Much less harmful	4.9 (3.8-6.4)	5.2 (3.9-6.9)	<.001
Less harmful	27.5 (25.1-30.1)	23.0 (20.6-25.5)	
About the same level	35.9 (33.1-38.8)	41.5 (38.6-44.3)	
More harmful	2.7 (2.0-3.7)	3.3 (2.5-4.4)	
Much more harmful	1.0 (0.5-1.9)	3.5 (2.5-4.8)	
Do not know	27.9 (25.4-30.6)	23.6 (21.2-26.3)	
Current smokers, No.	1171	1139	
Much less harmful	8.0 (6.2-10.4)	7.1 (5.3-9.4)	.001
Less harmful	25.5 (22.4-28.8)	23.5 (20.6-26.7)	
About the same level	33.9 (30.5-37.4)	39.3 (35.7-43.1)	
More harmful	3.7 (2.6-5.2)	5.2 (3.8-7.0)	
Much more harmful	1.8 (1.1-2.9)	4.4 (3.1-6.4)	
Do not know	27.1 (23.9-30.5)	20.5 (17.7-23.6)	
Current ENDS users, No.	411	460	
Much less harmful	24.0 (19.3-29.3)	19.4 (15.3-24.3)	.35
Less harmful	37.7 (32.0-43.7)	39.3 (33.9-44.9)	
About the same level	20.0 (15.7-25.0)	26.1 (21.4-31.5)	
More harmful	4.2 (2.1-8.0)	3.6 (2.0-6.6)	
Much more harmful	1.2 (0.6-2.4)	1.6 (0.7-3.8)	
Do not know	13.1 (9.4-17.9)	9.9 (7.2-13.6)	
Dual cigarettes and ENDS users, No.^d^	245	228	
Much less harmful	18.9 (13.6-25.7)	19.5 (13.9-26.6)	.87
Less harmful	32.3 (25.1-40.4)	34.5 (27.2-42.7)	
About the same level	30.1 (23.2-38.1)	32.7 (24.9-41.5)	
More harmful	4.1 (1.9-8.5)	2.5 (1.0-6.2)	
Much more harmful	2.1 (0.9-4.5)	1.2 (0.3-4.4)	
Do not know	12.5 (7.8-19.5)	9.6 (6.1-14.8)	

Abbreviation: ENDS, electronic nicotine delivery systems.

^a^ All percentages are weighted.

^b^ Based on χ^2^ tests of association.

^c^ Total of never, former, and current smokers.

^d^ Not combined with current ENDS users or any smoker group.

## Discussion

This survey study including data from late 2018, before the reported lung disease outbreak and deaths associated with vaping of 2019,^5^ found an increase in the proportion of US adults who perceived ENDS to be equally or more harmful than cigarettes. Notably, an increase in perceiving ENDS as much more harmful than cigarettes was observed among cigarette smokers, which may influence their decision to try or switch to ENDS use. The increase in perceived risk of ENDS from 2017 to 2018 may be associated with media coverage^6^ and the political and regulatory response to the rise in youth ENDS use in 2018. Given the recent surge in reported cases of lung illness associated with vaping and the rapidly evolving evidence, the extent of public uncertainty about the comparative harm of ENDS and cigarettes may have increased since 2018. A limitation of this study is that perceived harm of ENDS vs cigarettes was measured by a general question that did not describe specific health risks associated with product use. Perceptions of harm pertaining to specific diseases may differ from overall risk perceptions associated with ENDS use. In addition, these data were collected before the current vaping-related illness outbreak, and any resulting changes in perception are not captured by this study. Continued monitoring of comparative risk perceptions is warranted.
